# Analysis of Reporting Pattern in Children Aged 7 to 14 Years with Traumatic Injuries to Permanent Teeth

**DOI:** 10.5005/jp-journals-10005-1048

**Published:** 2010-04-15

**Authors:** Amit Vanka, KS Ravi, NM Roshan, ND Shashikiran

**Affiliations:** 1 Professor, Department of Pedodontics, Peoples College of Dental Sciences, Bhopal, Madhya Pradesh, India; 2 Senior Lecturer, Department of Pedodontics, Peoples College of Dental Sciences, Bhopal, Madhya Pradesh, India; 3 Senior Lecturer, Department of Pedodontics, Peoples College of Dental Sciences, Bhopal, Madhya Pradesh, India; 4 Dean, Professor and Head, Department of Pedodontics, Peoples College of Dental Sciences, Bhopal, Madhya Pradesh, India

**Keywords:** Traumatic injuries, anterior teeth, permanent teeth.

## Abstract

**Aim:**

To analyze the pattern of traumatic injuries to permanent anterior teeth reporting to the dental department with regards to age, gender, cause, proportion of different types of injury and time of reporting.

**Materials and Methods:**

Children aged 7 to 14 years with trauma or related sequelae were included. The data was collected retrospectively on the basis of case history, clinical findings, radiographs and vitality tests. Ellis’ classification was used to record injuries to anterior teeth.

**Results:**

Boys had more injuries with the highest injuries at 12 years. Various causes of trauma included Falls, RTA, hits by object/person and bicycle related. The most common injury reported were cases of Ellis’ class IV (50.7%) and the maxillary central incisors being the teeth most frequently involved (75%). The time lapsed after injury was more than 1 year in 42.8% cases and 62% cases reported with complications.

**Conclusion:**

Our findings suggest that a large number of cases reported in the age group 10 to 13 years with Class IV Ellis’ fracture largely when symptoms appear. Reporting was delayed by more than a year in several cases, the barriers for which need to be analyzed, to develop strategies regarding prevention of traumatic injuries and their consequences.

## INTRODUCTION

Orofacial trauma is among the most prominent oral health problems affecting children in developing countries.^1^ While diseases such as dental caries and periodontal disease have been given due importance and are still considered to be the most significant oral health problems worldwide, trauma to the anterior teeth with the underlying esthetic, psychosocial, functional, and therapeutic problems adversely affect an individual’s quality of life.^2^

Thus traumatic injuries to the anterior teeth pose a tremendous challenge to the dental profession, both in terms of treating the sequelae of injuries and preventing their occurrence. While prevalence of traumatic injuries is extensively researched, few studies exist on the reporting of such injuries, especially so in India. The goal of prevention requires reaching out at the community level to those particularly susceptible to trauma. This in turn necessitates accumulation of data as to the prevalence of traumatic in-juries to teeth and the associated factors such as the gender, tooth/teeth frequently affected, causes of trauma and others. The aptitude and priority accorded to such injuries can also be gauged, from the time lag occurring between injuries and reporting. Such studies can thus also be considered as tools for the implementation of effective preventive and educative strategies against fractures.

## AIM

To analyze the pattern of traumatic injuries to permanent anterior teeth reporting to the dental department with regards to age, gender, cause, proportion, of different types of injury and time of reporting.

## MATERIALS AND METHODS

A total of 202 subjects reporting to the department over a period of 18 months were included in the study group. The children selected were from 7 to 14 years by the time they had reported to the dental clinic. Subjects reporting with the chief complaint of trauma, its related complications such as pain, swelling, and discoloration of tooth or unesthetic appearance were included in the study. Cases of trauma which reported with chief complaint other than that of trauma or related sequelae, but were detected on routine examination, were also included. The data collection was done retrospectively. Ellis’ classification was used to record injuries.

Standardized procedures and the universal infection control precautions were followed during examination. Case history was used to determine the cause of trauma. Time lapsed after injury was also enquired and in the event that the cause could not be recollected, time lapse was considered from the time the first symptoms appeared. Intraoral periapical (IOPA) X-rays, vitality tests were used where ever they were deemed necessary. Data was subjected to descriptive and statistical analyses using SPSS for windows statistical software package Version 10.0. The One Sample Kolmogorov-Smirnov Test, with level of significance at 1% being defined as statistically significant was used. Chi-square test was used with 1% and 5% being defined as statistically significant.

## RESULTS

**Fig. 1:**
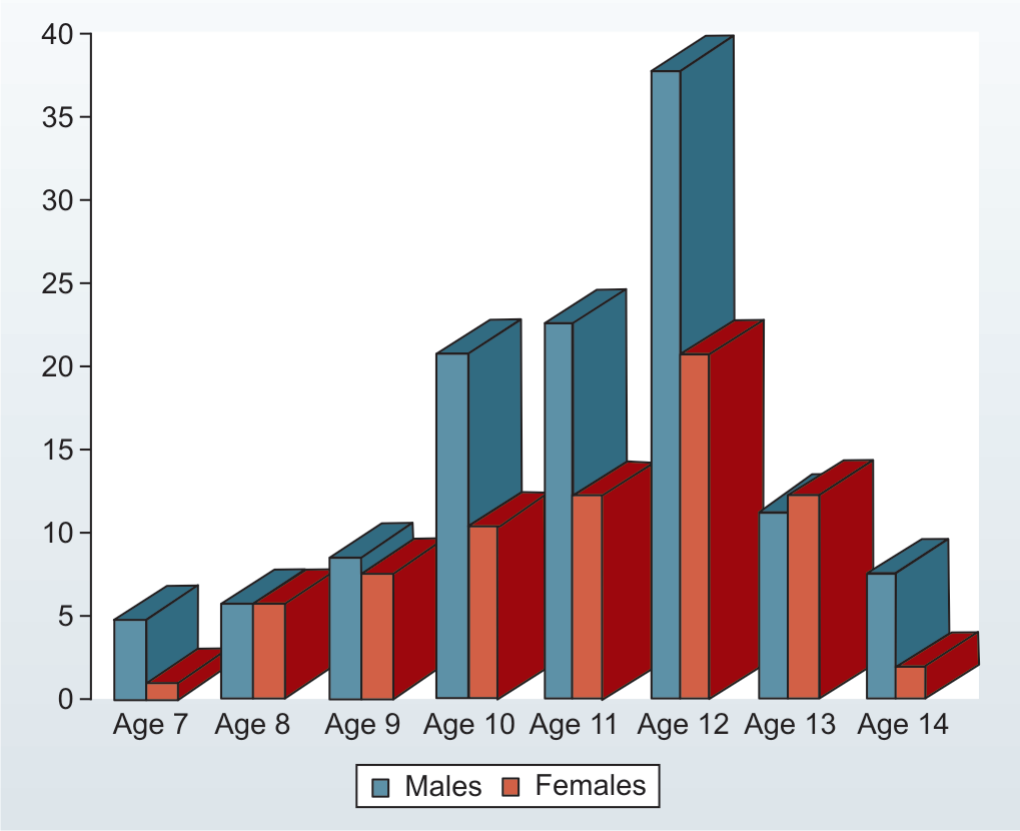
Distribution of patients according to age and sex

**Fig. 2:**
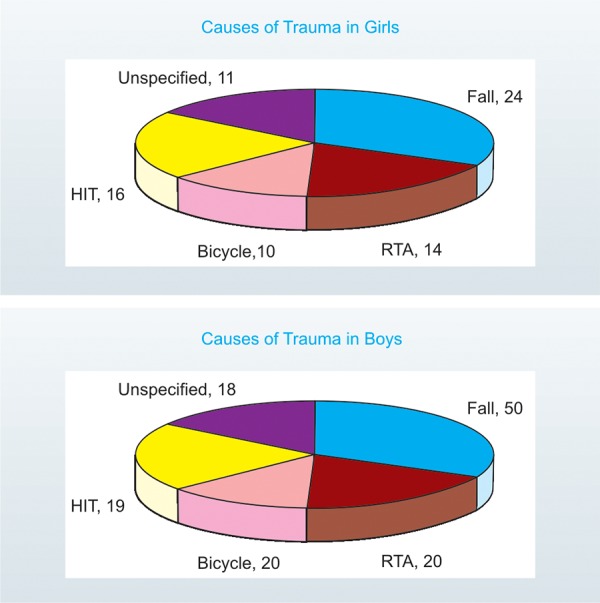
Causes of trauma in boys and girls

**Fig. 3:**
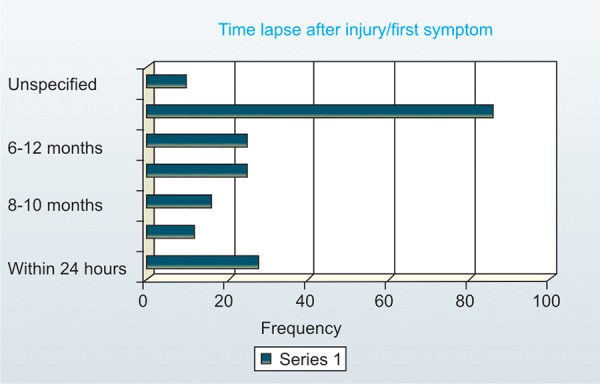
Distribution of cases according to time lapsed before reporting

1. A total of 202 subjects were deemed fit to be included in the study with 304 teeth being affected. The male female ratio was 1.7:1.0, with the highest frequency of trauma cases reported between 10 to 13 years. Injuries peaked at 12 years (29.3%) followed by 11 years (18.4%) (Fig. 1). There was no significant difference between boys and girls for the different ages.
2. *Cause of trauma:* 14.4 % cases did not specify or could not recollect any history of injury. Among the various causes of injury, fall was the most common type constituting 36.6% of the cases, followed by Hit by object or person (17.3%), RTA (16.9%), bicycle (14.8%). The level of significance at 1%, was highly significant: K-S (Z) = 7.528 indicating the variation in the data is real. There was no significant difference between girls and boys regarding causes of trauma (Fig. 2).
3. *Number of injuries per person:* The average number of injuries per person was 1.50. Out of 202 cases,
  - 121 (60%) children had 1 traumatized tooth;
  - 63 (31%) had 2 traumatized teeth;
  - 18 (9%) children had 3 traumatized teeth.
4. *Tooth commonly involved:* The tooth most frequently involved was the right maxillary central incisor constituting 118 cases followed by the left which was involved in 110. Together they formed 75% of the total cases (Table 1).
5. *Trauma scores:* Highest number of cases reported with Ellis class IV (154) followed by those with class II (77). The level of significance at 1%, was highly significant with: K-S (Z) = 7.723.
6. *Time elapsed after injury:* Out of the cases where the cause of injury was elicited or a history of symptoms existed 86 patients (42.6%) reported after 1 year followed by 28 individuals reporting within 24 hours (13.9%) (Fig. 3). No significant difference for reporting time was found between boys and girls.
  The time elapsed for Ellis class II and IV, was found to be not significant at*p* > 0.01 (Table 2).
7. *Reporting with or without symptoms:* Out of the cases reporting with chief complaint of trauma, 62% of the cases had complications such as pain, swelling and mobility while 38% were asymptomatic, concerned about esthetics.

**Table 1:** **Table 1:** Distribution of injuries according to tooth number

*Tooth*		*Class I*		*Class II*		*Class III*		*Class IV*		*Class V*		*Class VI*		*Class VII*		*Total (%)*	
11		15		32		8		58		3		0		2		118 (38.8%)	
12		2		3		1		5		3		0		0		14 (4.6%)	
21		10		29		4		64		2		0		1		110 (36.1%)	
22		7		2		1		11		3		0		0		24 (7.9%)	
31		2		5		1		7		3		0		0		18 (5.9%)	
32		0		2		0		2		1		0		0		5 (1.6%)	
41		2		3		0		6		1		0		0		12 (3.9%)	
42		0		1		0		1		0		0		1		3 (1.0%)	
Total (%)		38 (12.5%)		77 (25.3%)		15 (4.9%)		154 (50.7%)		16 (5.3%)		0 (0%)		4 (1.3%)		304 (100%)	

## DISCUSSION

Traumatic injuries to the teeth constitute the most serious dental condition experienced by children.^2^ Since the majority of these are preventable, there is an emerging consensus that traumatic dental injuries constitute a major public health problem and that oral health promotion programs to prevent such injuries are necessary in communities, where, they are either frequent or severe.^3^

There has been an array of classifications used in prevalence studies. In the current study, Ellis’ classification of dental trauma was used.^4^ Retrospective studies on dental trauma inherently have certain drawbacks. Soft tissue injuries and injuries to periodontal tissues cannot be recorded if the patient reports much later than the date of injury. Thus concussion and subluxation cases were excluded. History given by patient cannot be completely relied upon as the event has occurred sometime back.^5^

### 1. Gender Ratio

In the present study boys-girl ratio was 1.7:1.0. Most international surveys in the past unanimously agree that males experience significantly more dental trauma to the permanent dentition than females. The boy: girl ratio has ranged from 1.3:1 to 2.7:1^6-9^ and the findings in this study are comparable to those cited above.

Boys are known to be involved in physical games. Another reason often quoted is the indulgence in more violent behavior by boys and could be a contributory factor for more injuries to boys.

There have been exceptions, however, such as the one conducted by Garcia-Godoy^10^ wherein a male: female ratio of 0.9:1.0 was observed.

### 2. Age of Trauma

The findings of the current study are that most of the children were in the age group of 10 to 13 years. This range is consistent with the findings of some studies,^9,11^ while others report it on the lower side of 8 to 9 years.^8,12^ Even though the age group affected in this study appears to correlate well with previous studies, the effect of time lapse between injury and reporting has not been clarified by many. Thus, whereas the trauma reporting was maximum at 12 years, the injuries would have probably occurred at an age at least 1 to 1.5 years earlier.

**Table 2:** **Table 2:** Comparison of class II and IV with time lapsed after injury*

		*Within* *1 month*		*1-6 months*		*6 months* *1 year*		*More than* *1 year*		*total*	
Ellis’ II		9		9		6		11		35	
Ellis ’IV		9		10		5		75		99	
Total		18		19		11		86		134	

*Chi-square = 20.36 > 0.01

### 3. Cause of Trauma

The most common cause of injury was due to falls, which has been supported by several other studies in this regard.^8,13-15^ Falls occurred while playing, tripping, chasing, falling off the bed during sleep and while climbing trees. Road traffic accidents, Hits by an object or person were also common and could be associated with sports or fighting, though in 3 cases at least, the suspicion of child abuse could not be ruled out. Bicycle injuries were next including falling of a bicycle. Since the bicycle continues to be a frequently used mode of transport in these parts, learning the same may be associated with injury. Unfortunately even the use of helmets has not been found to be effective in reducing dental trauma as compared to head injuries and facial trauma,^16^ unless the design of the helmet is modified.

### 4. Number of Injuries per Person

In this study, on an average, injuries per person was 1.50, with majority of cases (60%) having only one tooth involved. The number of injuries per patient has varied from between 1.1 and 2.0^5^ and the findings in the current study fall in that range. The results for single or multiple teeth have been equivocal. Whereas, hospital based studies encountered more cases with multiple teeth involved,^17^ those in institutes or private practices have found only one tooth most commonly involved.^18,19^

### 5. Tooth Commonly Involved

The right maxillary central incisor was the most common tooth involved which accounted for 39% of the injuries followed by the left maxillary central incisor with 36%. Together the maxillary central incisors accounted for 75% of the injuries. These findings are similar to those previously reported.^17-23^ As the position of these teeth in the oral cavity predisposes these teeth to frequent trauma, current findings were not surprising. Shulman and Peterson^20^ concluded that after nullifying the effect of age, gender and ethnicity, the odds of trauma increased with increase in overjet, explaining at least some of the cases in the current study.

### 6. Type of Injury/Score

The number of devitalized teeth (Ellis class IV) is unusually high in the current study and is in contrast to studies which have reported that the injuries to enamel only^21,22^ or enamel and dentin^23^ were most frequently observed. Traumatized teeth, left untreated, are known to have an increased tendency to show irreversible pulpal damage^11^ and the increased number of class IV cases reporting later than 1 year is demonstrated in the current study as well. A small percentage (4.9%) of cases met the criteria for apexogenesis, i.e. they reported within 24 hours with a traumatic pulp exposure allowing a calcium hydroxide pulpotomy to be done. This is an area that can show significant improvement by oral health education.

### 7. Time Lapse between Injury and Reporting

The reporting time after injury was most frequently a year or more after the injury in the current study. A study done in the department of pediatric dentistry, Beijing^8^ found that 41.21% of children reported within 2 to 24 hours while several others within 1 to 7 days. Another study in Belgium^24^ reported that 79% of children reported within 2 days of injury. Reporting late points to the low level of priority accorded to such injuries. This probably indicates the lack of knowledge, both, regarding preventive methods, as well as the sequalae of untreated traumatic injuries and their treatment modalities.

### 8. Symptomatic *vs* Asymptomatic

Children were brought to the clinic largely with symptoms. Probably the subjects were taken or reported to the dentist only when the symptoms of trauma such as swelling, discoloration and pain manifested, another indication to the neglect of traumatic injuries.

## CONCLUSION

Our findings suggest that there are an increased number of cases reporting in the ages 10 to 13 years, with a sizable number of them being boys. Many cases reported more than a year after trauma, frequently when the symptoms appear. Half the teeth examined were seen to be in a devitalized condition. The causes of trauma are the same ones frequently encountered in the literature. The barriers for late reporting need to be analyzed.

## CLINICAL SIGNIFICANCE

Strategies at community level should include education regarding the various modalities of avoiding such injuries. The population prone to such injuries should also be made aware of the consequences of untreated traumatic injuries and encouraged to report to the dentist at the earliest.

## References

[1] World Health Organisation/AFRO. Oral health. Epidemiology [Internet]. July 2001 Available from:. http://www.afro.who.int/oralhealthepidemiology.html.

[2] Andreasen JO., Andreasen FM., Andersson L. (1994). Textbook and atlas of traumatic injuries to the teeth.

[3] Daly B., Watt RG., Batchelor P (2002). Essential dental public health.

[4] Ellis RG (1970). The classification and treatment of injuries to the teeth of children.

[5] Bastone EB, Freer TJ, McNamara JR (2000). Epidemiology of dental trauma: a review of the literature.. Aust Dent J.

[6] Petti S, Tarsitani G (1996). Traumatic injuries to anterior teeth in Italian schoolchildren: prevalence and risk factors.. Endod Dent Traumatol.

[7] Saroglu I, Sonmez H (2002). The prevalence of traumatic injuries treated in the pedodontic clinic of Ankara University, Turkey, during 18 months.. Dent Traumatol.

[8] Lihong G, Chen J, Zhao Y, Xia B, Kimura M (2005). Analysis of traumatic injuries to 886 permanent anterior teeth.. J Hard Tissue Biol.

[9] Zerman N, Cavalleri G (1993). Traumatic injuries to permanent incisors.. Endod Dent Traumatol.

[10] Garcia-Godoy FM (1984). Prevalence and distribution of traumatic injuries to the permanent teeth of Dominican children from private schools.. Community Dent Oral Epidemiol.

[11] Oulis CJ, Berdouses ED (1996). Dental injuries of permanent teeth treated in private practice in Athens.. Endod Dent Traumatol.

[12] Caliskan MK, Türkün M (1995). Clinical investigation of traumatic injuries of permanent incisors in Izmir, Turkey.. Endod Dent Traumatol.

[13] Rocha MJ, Cardoso M (2001). Traumatized permanent teeth in Brazilian children at the Federal University of Santa Catarina, Brazil.. Dent Traumatol.

[14] Glendor U (2009). Aetiology and risk factors related to traumatic dental injuries- a review of the literature.. Dent Traumatol.

[15] Perez R, Berkowitz R, McIlveen L, Forrester D (1991). Dental trauma in children: A survey.. Endod Dent Traumatol.

[16] Chapman HR, Curran AL (2004). Bicycle helmets-does the dental profession have a role in promoting their use?. Br Dent J.

[17] Galea H (1984). An investigation of dental injuries treated in an acute care general hospital.. J Am Dent Assoc.

[18] Stockwell AJ (1988). Incidence of dental trauma in the Western Australian School Dental Service.. Community Dent Oral Epidemiol.

[19] Davis GT, Knott SC (1984). Dental trauma in Australia.. Aust Dent J.

[20] Shulman JD, Peterson J (2004). The association between incisor trauma and occlusal characteristics in individuals 8-50 years of age.. Dent Traumatol.

[21] Pradeep ST (2007). The prevalence of anterior teeth fracture and its relation to malocclusion in 12 and 15 year old school children.. J Oral Health Comm Dent.

[22] Locker D (2005). Prevalence of traumatic dental injury in grade 8 children in six Ontario communities.. Can J Public Health.

[23] Baldava P, Anup N (2007). Risk factors for traumatic dental injuries in an adolescent male population in India.. J Contemp Dent Pract.

[24] Shayegan A, Maertelaer V, Abbeele AV (2007). The prevalence of traumatic dental injuries: a 24-month survey.. J Dent Child (Chic).

